# Analysis of incidental findings in Qatar genome participants reveals novel functional variants in *LMNA* and *DSP*

**DOI:** 10.1093/hmg/ddac073

**Published:** 2022-03-26

**Authors:** Amal Elfatih, Sahar I Da’as, Doua Abdelrahman, Hamdi Mbarek, Idris Mohammed, Waseem Hasan, Khalid A Fakhro, for the The Qatar Genome Program Research Consortium, Said I Ismail, Wadha Al-Muftah, Radja Badji, Hamdi Mbarek, Dima Darwish, Tasnim Fadl, Heba Yasin, Maryem Ennaifar, Rania Abdel-latif, Fatima Alkuwari, Muhammad Alvi, Yasser Al Sarraj, Chadi Saad, Asmaa Althani, Eleni Fthenou, Fatima Qafoud, Eiman Alkhayat, Nahla Afifi, Sara Tomei, Wei Liu, Stephan Lorenz, Najeeb Syed, Hakeem Almabrazi, Fazulur Rehaman Vempalli, Ramzi Temanni, Tariq Abu Saqri, Mohammed Husen Khatib, Mehshad Hamza, Tariq Abu Zaid, Ahmed El Khouly, Tushar Pathare, Shafeeq Poolat, Rashid Al-Ali, Omar M E Albagha, Souhaila Al-Khodor, Mashael Alshafai, Ramin Badii, Lotfi Chouchane, Xavier Estivill, Khalid A Fakhro, Hamdi Mbarek, Younes Mokrab, Jithesh V Puthen, Karsten Suhre, Zohreh Tatari, Xavier Estivill, Borbala Mifsud

**Affiliations:** College of Health and Life Sciences, Hamad Bin Khalifa University, Doha, Qatar; Department of Human Genetics, Sidra Medicine, Doha, Qatar; College of Health and Life Sciences, Hamad Bin Khalifa University, Doha, Qatar; Department of Human Genetics, Sidra Medicine, Doha, Qatar; Department of Human Genetics, Sidra Medicine, Doha, Qatar; Qatar Genome Programme, Qatar Foundation Research, Development and Innovation, Qatar Foundation, Doha, Qatar; College of Health and Life Sciences, Hamad Bin Khalifa University, Doha, Qatar; Department of Human Genetics, Sidra Medicine, Doha, Qatar; College of Health and Life Sciences, Hamad Bin Khalifa University, Doha, Qatar; Department of Human Genetics, Sidra Medicine, Doha, Qatar; Quantitative Genomic Medicine Laboratories (qGenomics), Barcelona, Spain; College of Health and Life Sciences, Hamad Bin Khalifa University, Doha, Qatar; William Harvey Research Institute, Queen Mary University London, London EC1M 6BQ, UK

## Abstract

In order to report clinically actionable incidental findings in genetic testing, the American College of Medical Genetics and Genomics (ACMG) recommended the evaluation of variants in 59 genes associated with highly penetrant mutations. However, there is a lack of epidemiological data on medically actionable rare variants in these genes in Arab populations. We used whole genome sequencing data from 6045 participants from the Qatar Genome Programme and integrated it with phenotypic data collected by the Qatar Biobank. We identified novel putative pathogenic variants in the 59 ACMG genes by filtering previously unrecorded variants based on computational prediction of pathogenicity, variant rarity and segregation evidence. We assessed the phenotypic associations of candidate variants in genes linked to cardiovascular diseases. Finally, we used a zebrafish knockdown and synthetic human mRNA co-injection assay to functionally characterize two of these novel variants. We assessed the zebrafish cardiac function in terms of heart rate, rhythm and hemodynamics, as well as the heart structure. We identified 52 492 novel variants, which have not been reported in global and disease-specific databases. A total of 74 novel variants were selected with potentially pathogenic effect. We prioritized two novel cardiovascular variants, *DSP* c.1841A > G (p.Asp614Gly) and *LMNA* c.326 T > G (p.Val109Gly) for functional characterization. Our results showed that both variants resulted in abnormal zebrafish heart rate, rhythm and structure. This study highlights medically actionable variants that are specific to the Middle Eastern Qatari population.

## Introduction

The American College of Medical Genetics and Genomics (ACMG) recommends reporting medically actionable pathogenic variants in 59 genes when ordering clinical genomic testing (1,2). Individuals carrying pathogenic variants in these 59 genes are at high risk to develop highly penetrant diseases in the future. Early detection and screening can be beneficial for them to receive early medical intervention and ameliorate the clinical presentation. The frequency of these medically actionable variants had been assessed in global population studies (3–7). However, there is a lack of data on the frequency of medically actionable variants in ethnic groups that are not well represented in global sequencing efforts, such as the Middle Eastern population. Moreover, the frequency of rare medically actionable variants in the Qatari population is predicted to be high due to the founder effect and high consanguinity rate in Qatar (8–12). The WGS and comprehensive phenotypic data collected by the Qatar Genome Programme (QGP) and the Qatar Biobank (QBB) allows us to investigate the prevalence of medically actionable variants in the Qatari population and to identify novel, potentially pathogenic variants in clinically relevant genes.

In this study, we analyzed the novel variants in the 59 ACMG genes using 6045 WGS data from the pilot phase of the QGP. These variants were not reported previously in any database. We assessed the clinical phenotypic data associated with cardiovascular diseases for the genotype positive QGP participants in order to identify putative novel variants that could cause cardiovascular diseases.

Zebrafish is a very useful *in vivo* cardiac disease model to characterize the pathogenicity of predicted pathogenic novel variants owing to the transparency of zebrafish during early development, and their ability to tolerate severe cardiac anomalies (13–15). Therefore, we utilized the zebrafish model to validate *in vivo* the pathogenicity and the phenotype–genotype association for two novel cardiovascular variants, *DSP* c.1841A > G (p.Asp614Gly) and *LMNA* c.326 T > G (p.Val109Gly). *DSP* and *LMNA* pathogenic genetic variants are known to cause arrhythmogenic and dilated cardiomyopathy or laminopathy, respectively. We used knockdown of the zebrafish orthologs (*dspa*/*dspb* and *lmna*) and co-injected zebrafish embryos with synthetic RNA of the human *DSP* and *LMNA* to mimic the novel genetic variants.

## Results

### Potentially pathogenic novel ACMG variants identified in the QGP cohort

**Figure 1:**
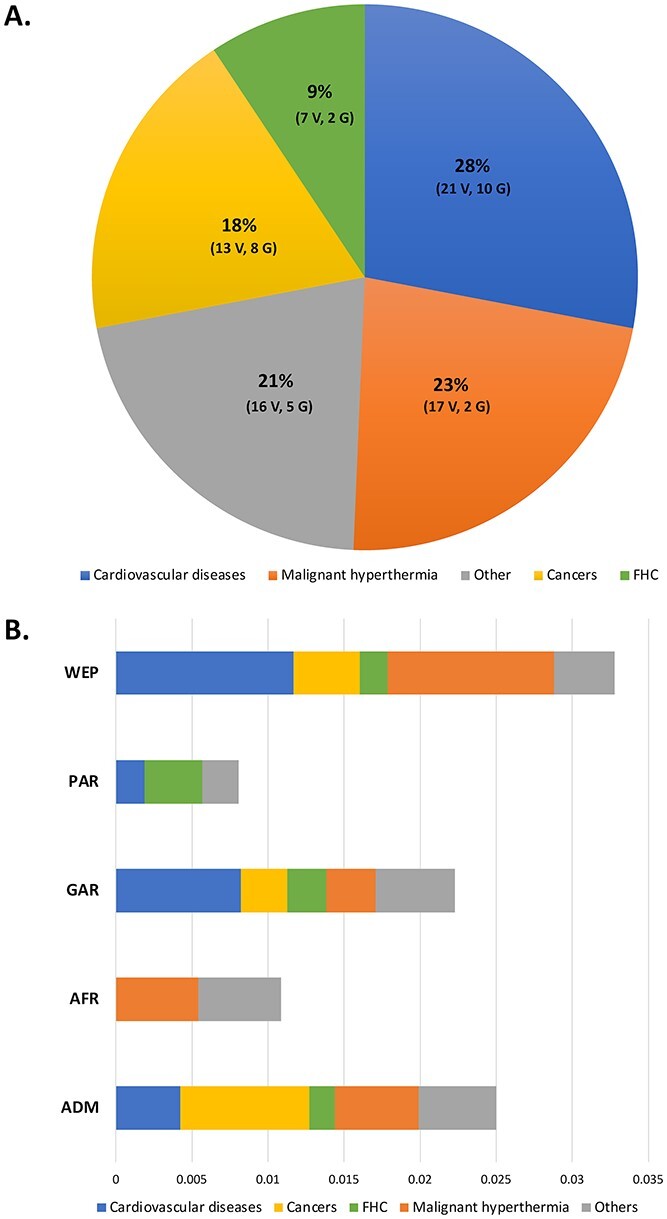
Distribution of ACMG novel variants among disease categories and QGP sub-population. (**A**) Frequency of 74 filtered novel ACMG variants based on different disease categories. (**B**) Distribution of novel ACMG variants in the QGP sub-populations. Abbreviations: FHC; Familial hypercholesteremia, GAR; General Arabs, PAR; Peninsular Arabs, WEP; Arabs of Western Eurasia and Persia, AFR; Arabs with African ancestry, ADM; Admixed, V; variants, G; genes.

The number of variants identified at each stage of our filtration process is shown in the summarized flow chart (Supplementary Material, Fig. S1). We retrieved 148 285 variants in 59 ACMG genes. After filtering out the variants present in any of dbSNP, gnomAD, TOPMed, 1000 Genome Project, ExAC, The Greater Middle East (GME) Variome Project, The Human Gene Mutation Database (HGMD) or ClinVar; we found 325 novel variants with Combined Annotation Dependent Depletion (CADD) score ≥ 20 and the Genomic Evolutionary Rate Profiling (GERP) ≥3. By applying our filtering criteria of minor allele frequency (MAF), single allele count, unrelated alleles, variant impact and effect, a total of 74 novel variants had been selected (Supplementary Material, Table S1). The 74 identified novel variants were in 26 of the 59 ACMG genes. These 26 genes are known to be associated with five disease categories, cardiovascular diseases, cancers, familial hypercholesteremia (MIM 143890), Malignant hyperthermia (MIM 145600) and others (Supplementary Material, Table S2). The highest frequency of novel variants among the 74 potentially pathogenic variants were found in genes associated with cardiovascular diseases (28%, 21 variants, 10 genes) followed by variants associated with Malignant hyperthermia (23%, 17 variants, 2 genes) (Fig. 1A).

We found that five of the six subpopulations present in Qatar have a specific set of candidates pathogenic ACMG variants, which predispose individuals in those subpopulations for specific diseases. The 74 novel variants were distributed across the five QGP sub-populations with the highest allele frequency of 0.033 in Arabs of Western Eurasia and Persia (WEP) followed by 0.025 in Admixed (ADM), 0.022 in General Arabs (GAR), 0.010 in Arabs with African ancestry (AFR) and 0.008 in Peninsular Arabs (PAR). We did not detect any novel ACMG variants in Arabs with South Asian ancestry (SAS). Variants associated with all five disease categories were found in WEP, ADM and GAR (Fig. 1B and Table 1). Although we observed high frequency of variants associated with cardiovascular diseases among different sub-populations, we did not detect any variant associated with cardiovascular diseases in the AFR subpopulation**.**

**Table 1:** Sub-population distribution of novel ACMG variants in associated disease categories

	**ADM AF** **(*n* = 1180)**	**AFR AF** **(*n* = 92)**	**GAR AF** **(*n* = 2311)**	**PAR AF** **(*n* = 1052)**	**WEP AF** **(*n* = 1372)**	**SAS AF** **(*n* = 38)**
**ACMG genotype positive participants frequency**	0.025000023	0.01086956	0.02228472	0.008079848	0.03279883	0
**Cardiovascular diseases**	0.004237294	0	0.008221547	0.001901141	0.011661803	0
**Cancers**	0.00847459	0	0.003028989	0	0.004373177	0
**Familial hypercholesteremia**	0.001694916	0	0.002596279	0.003802281	0.001822161	0
**Malignant hyperthermia**	0.005508472	0.00543478	0.00324535	0	0.01093294	0
**Others**	0.005084751	0.00543478	0.005192555	0.002376426	0.004008749	0

Abbreviations: GAR: General Arabs, PAR: Peninsular Arabs, WEP: Arabs of Western Eurasia and Persia, SAS: Arabs with South Asian ancestry, AFR: Arabs with African ancestry, ADM: Admixed, *n*: number of participants. AF: allele frequency.

### Phenotypic analysis of novel variants associated with cardiovascular diseases

Seven variants in six genes were found in individuals with cardiovascular disease phenotypes, *SCN5A* c.2309C > T (p.Ala770Val)*, LMNA* c.326 T > G (p.Val109Gly), *MYBPC3* c.1021G > T (p.Gly341Cys), *TPM1* c.674A > G (p.Tyr225Cys), *DSP* c.1841A > G (p.Asp614Gly) and c.7604A > G (p.Asp2535Gly), and *RYR2* c.13919 T > C (p.Val4640Ala). Detailed phenotypic analysis of these variants is shown in Supplementary Material, Table S3. Two of these variants, *DSP* c.1841A > G (p.Asp614Gly) and *LMNA* c.326 T > G (p.Val109Gly), were significantly associated with cardiovascular disease in our cohort, based on the criteria we used to define cardiovascular disease phenotypes. We selected these two variants for functional validation in zebrafish.

### Cardiovascular phenotype of *DSP* c.1841A > G (p.Asp614Gly)

We found 11 QGP subjects carrying the *DSP* c.1841A > G (p.Asp614Gly) novel genetic variant. The identified variant has a CADD score of 24.5 and GERP of 5.79. Our established criteria for QBB phenotypic data identified association with cardiovascular phenotypes. Three participants out of the 11 genotype positive participants had an abnormal electrocardiogram (ECG), and one participant had borderline ECG with chest pain (*P* = 0.0290, and OR = 6.619). Five participants reported parental history of heart disease (*P* = 0.3197, and OR = 1.984). Of note, one participant self-reported a personal history of angina.

### Cardiovascular phenotype of *LMNA* c.326 T > G (p.Val109Gly)

Our analysis identified nine QGP subjects carrying the *LMNA* c.326 T > G (p.Val109Gly) novel genetic variant. The variant has a CADD score of 25.9 and GERP of 4.9. Two genotype positive participants out of the nine had abnormal ECG recordings, and three participants had a borderline ECG profile with chest pain (*P* = 0.0364, and OR = 4.138). Six participants reported to have parental history of heart disease (*P* = 0.0238, OR = 4.755).

### *In silico* analysis of *DSP* c.1841A > G (p.Asp614Gly) and *LMNA* c.326 T > G (p.Val109Gly)

To predict the impact of the QGP identified novel *DSP* and *LMNA* variants, we performed *in silico* prediction of these missense genetic variations using MutationTaster and I-Mutant2.0. For the c.1841A > G (p.Asp614Gly) variant in the *DSP* gene, MutationTaster showed that the position is conserved (phastCons 0.947, phyloP 1.599), the variant is moderately conservative (Grantham 94) and predicted that the amino acid change was likely to be disease causing by altering the protein features. The variant marginally increased the strength of a splice acceptor site, but the length of the resulting protein was predicted to be normal.

The *LMNA* c.326 T > G (p.Val109Gly) variant showed even higher level of conservation (phastCons 1, phyloP 2.725) and higher evolutionary dissimilarity (Grantham 109). MutationTaster also indicated a possible effect of this variant on splice sites; however, the length of the resulting protein is expected to be normal. This variant is predicted to be disease causing by affecting protein features.

We assessed the change in protein stability due to these *DSP* and *LMNA* variants using I-Mutant2.0. Both the *DSP* c.1841A > G (p.Asp614Gly) and *LMNA* c.326 T > G (p.Val109Gly) variants were predicted to decrease protein stability at pH 7.0 and 25°C with a reliability index of 6 and 9, respectively. In order to better understand the consequences of the *in silico* predicted disease-causing effect on cardiac structure and function, we utilized the zebrafish model.

Multiple sequence alignment between the human DSP protein (NP_001008844.1) and the human LMNA protein (NP_733821.1), and their orthologs in other species, showed the conserved amino acid location of the novel *DSP* and *LMNA* missense variants. The *LMNA* variant location is conserved across all studied species except chicken, while the *DSP* variant location is conserved in rodents and chicken but differs in zebrafish (Supplementary Material, Fig. S2). However, the zebrafish orthologs for both genes showed high sequence similarity, 71.4% with the human DSP and 85.1% with the human LMNA protein (16).

### Characterization of predicted pathogenic *DSP* and *LMNA* missense variants in zebrafish

We sought to employ the zebrafish model to functionally validate the novel variants’ effect on cardiac structure and function. To mimic the human *DSP* and *LMNA* genetic variants in zebrafish, we co-injected human synthetic mRNA with morpholinos (MO) designed to knockdown the endogenous zebrafish orthologs. The *in vitro* transcribed wild type (WT) or variant carrying (Variant) human RNA was co-injected with either a *dspa* + *dspb* MO mix or *lmna* MO (Supplementary Material, Fig. S2). The knockdown of endogenous zebrafish genes by *dspa* + *dspb* (*dspa*/*b* MO) and *lmna* MO was confirmed by RT-qPCR (Supplementary Material, Fig. S3). The expression level of *dspa, dspb* and *lmna* was 7%, 8.3% and 3.1% in MO knockdown compared to uninjected controls (*P* < 0.0001).

For the *DSP* zebrafish model, both the novel *DSP* variant (MO + DSP^Variant^) and the knockdown alone (*dspa/b* MO) showed a negative effect on the survival rate (58%, 59%), respectively, compared to the uninjected control groups (90%) (*P* < 0.0001). However, zebrafish expressing the human WT *DSP* (MO + DSP^WT^) showed no statistically significant difference in the survival rate (81%) when compared with the control. While for the *LMNA* model, the *LMNA* variant (MO + LMNA^Variant^), knockdown alone (*lmna* MO) and WT (MO + LMNA^WT^) were all found to have a negative effect on the survival rate (40%, 37%, 65%), respectively, compared to control group (90%) (*P* < 0.0001) (Supplementary Material, Fig. S4B). Both models had a negative effect on zebrafish development (Supplementary Material, Fig. S4C). We found that the proportion of the normally developed group for the MO + DSP^Variant^, *dspa/b* MO and MO + DSP^WT^ is lower (37.7%, 42.5%, 51.4%, respectively), compared to control group (100%) (p < 0.0001). Similarly, in the zebrafish *LMNA* model, the MO + LMNA^Variant^, the *lmna* MO and the MO + LMNA^WT^ had 28.4%, 34.4% and 22.8%, normally developed animals, respectively, compared to control group (100%) (*P* < 0.0001).

### Novel *DSP* variant results in bradycardia

**Figure 2:**
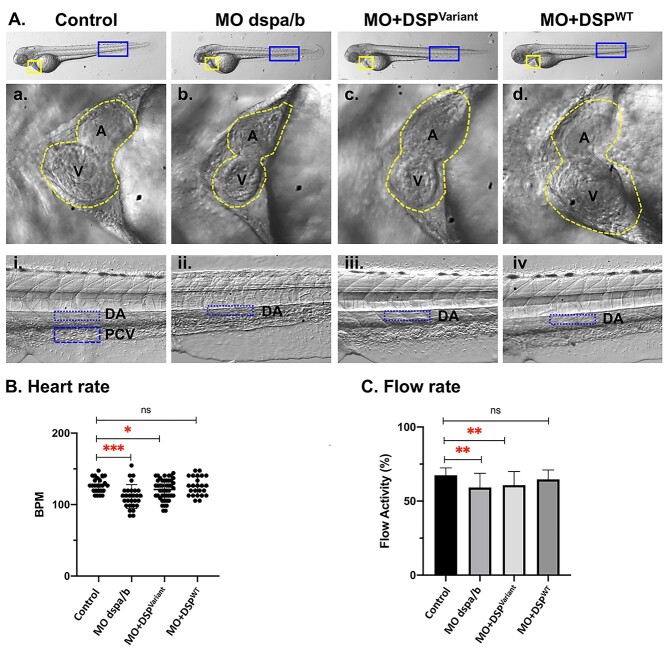
Zebrafish *DSP* Model Cardiac examination at 72 hours post fertilization (hpf). Modeling Qatar Genome Programme human *DSP* cardiac variant produced specific cardiac phenotype in the zebrafish model, representative heart images at 72 hours post-fertilization (hpf): (**A**) Zebrafish larvae at 72 hpf showing the two chambered heart (yellow square) and dorsal aorta flow rate (blue rectangle) images at 32X magnification. Representative images of the heart chambers; atrium (a) and ventricle (v) (traced with yellow dotted line) in the zebrafish experimental groups (control, Morpholino injected (MO *dspa/b*), human synthetic RNA of variant *DSP* c.1841A > G (p.Asp614Gly) (MO + DSP^Variant^) and wild-type (MO + DSP^WT^) co-injected with corresponding MO targeting the endogenous zebrafish transcript). (**B**) Heart rate for each individual fish was calculated as beats per minute (bpm) for the four experimental groups. Each dot represents an animal. Average heart rate of each group was compared to the control group. (**C**) The analysis of the vascular parameters was performed to calculate the blood flow rate within a selected area of the blood vessel (dorsal aorta). Average blood flow activity of each group was compared to the control group. Control (*n* = 25), *dspa/b* MO (*n* = 28), MO + DSP^Variant^ (*n* = 46), and MO + DSP^WT^ (*n* = 24). ns: not significant.

We chose to examine our established *DSP* c.1841A > G (p.Asp614Gly) zebrafish model at 72 hpf referring to the development of the cardiac conduction system in zebrafish (Fig. 2A) (17). We examined the zebrafish cardiac function for the different groups. We found that both the MO + DSP^Variant^ and the zebrafish knockdown (*dsp*a*/b* MO) significantly decreased the heart rate to an average of 121.1 beats per minute (bpm) and 111.2 bpm, respectively, compared to the control at 127.1 bpm (Fig. 2B and Supplementary Material, Fig. S5). While the MO + DSP^WT^ heart rate at 125.7 bpm was not significantly different from the control (Fig. 2B and Supplementary Material, Fig. S5).

### Zebrafish *DSP* model displays altered hemodynamics resulting in altered rhythm

Quantitation of the aortic blood flow activity in zebrafish was analyzed to investigate the hemodynamics across the examined zebrafish groups. Both groups of human *DSP* variant-expressing (MO + DSP^Variant^) and zebrafish knockdown alone (*dspa/b* MO) displayed a significantly altered blood flow activity. The percentage of the blood flow activity within the DA was significantly decreased with an average of 60.80% and 59.19% for both MO + DSP^Variant^, and the *dspa/b* MO, respectively, compared to the MO + DSP^WT^ and control zebrafish with an average of 64.70% and 67.49%, respectively (Fig. 2C).

**Figure 3:**
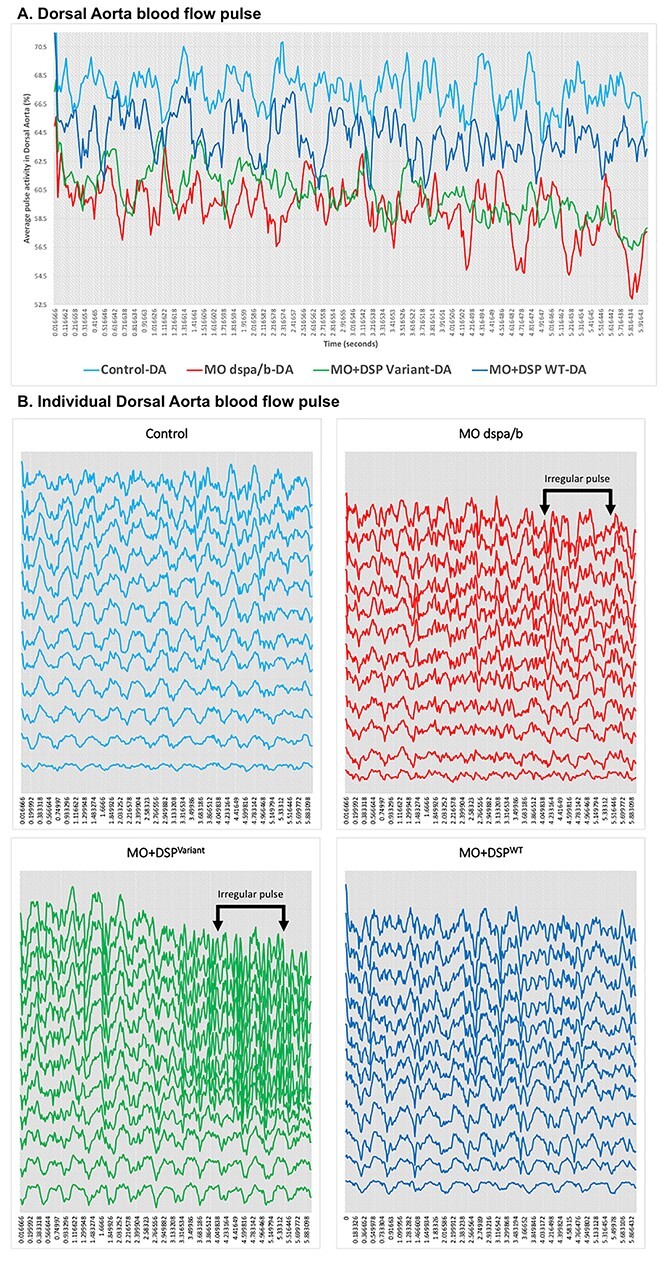
Zebrafish *DSP* model cardiac blood flow pulse in Dorsal Aorta. Modeling Qatar Genome Programme human *DSP* cardiac variant produced altered blood flow pulse in the dorsal aorta (DA) of the zebrafish model, representative charts at 72 hours post-fertilization (hpf): (**A**) Video recordings of high-speed acquisition of the blood flow (60 frames per second) were processed, a selected area of the DA with circulating red blood cells was used to calculate the cardiac blood flow pulse. A representative flow profiles chart for the analysis provides a color-coded pulse over time (6 s). (**B**) Representative individual pulse of analyzed data (*n* = 12 per group).

We assessed the heart rhythm pattern in the DA, the main artery that is directly connected to the heart chamber. The blood flow pattern in the DA is consistent with the rhythm of the heart (18,19). We found that the *DSP* genetic variant (MO + DSP^Variant^) caused abnormal cardiac rhythm. Both groups of MO + DSP^WT^ and control displayed similar, rhythmic blood flow patterns. In contrast, the MO + DSP^Variant^ and the zebrafish *dspa/b* MO groups presented with irregular DA blood flow patterns suggesting altered cardiac rhythms (Fig. 3).

### *LMNA* variant results in altered heart rate in zebrafish

**Figure 4:**
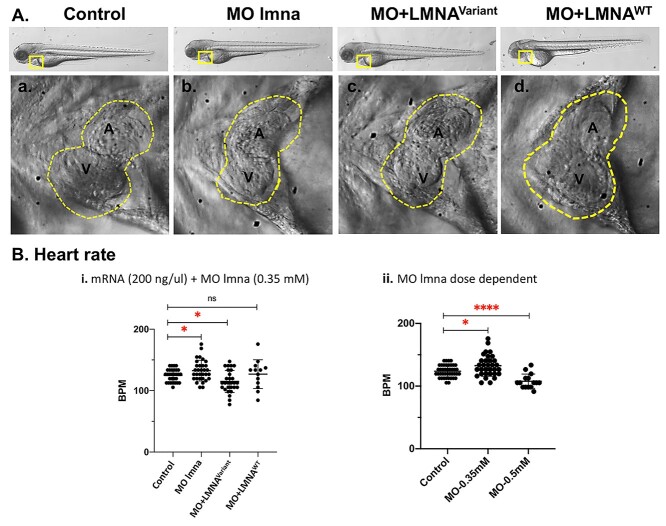
Zebrafish *LMNA* Model Cardiac examination at 72 hours post fertilization (hpf). Modeling Qatar Genome Programme human *LMNA* cardiac variant produced specific cardiac phenotype in the zebrafish model, representative heart images at 72 hpf: (**A**) Zebrafish larvae at 72 hpf showing the two chambered heart (yellow square), and dorsal aorta flow rate (blue rectangle). Zebrafish examined groups were control, Morpholino injected group (MO *lmna*), human synthetic RNA of variant *LMNA* c.326 T > G (p.Val109Gly) (MO + LMNA^Variant^) and wild-type (MO + LMNA^WT^) co-injected with corresponding MO targeting the endogenous zebrafish transcript. Representative images of the heart chambers; atrium (a) and ventricle (v) (traced with yellow dotted line). (**B**) Heart rate for each individual fish was calculated as beats per minute (bpm) for the four experimental groups. Each dot represents an animal. Average heart rate of each group was compared to the control group. i: Control (*n* = 30), *lmna* MO (*n* = 17), MO + LMNA^Variant^ (*n* = 28), and MO + LMNA^WT^ (*n* = 13). ii: Control (*n* = 42), MO_0.35 (*n* = 32) and MO_0.5 (*n* = 15). ns: not significant.

The *LMNA* variant (MO + LMNA^Variant^) showed a significant effect on the injected embryos’ survival rate. However, the surviving zebrafish’s hearts showed no overt global morphological alterations. The clinical manifestations of patients with cardiac laminopathy have a wide spectrum ranging between tachycardia, dilated cardiomyopathy, bradycardia and sudden cardiac death (20). The novel *LMNA* variant led to a significant decrease in the calculated heart rate (114.9 bpm) mimicking bradycardia (Fig. 4B). In comparison, the MO + LMNA^WT^ and control zebrafish groups calculated heart rates were at 126.8 bpm and 124.9 bpm, respectively. Interestingly, the *lmna* MO in zebrafish resulted in a significant increase of the calculated heart rate at 132.5 bpm mimicking tachycardia.

Further, to investigate the effect of the *lmna* zebrafish knockdown on cardiac function, we performed dose titration experiments. The zebrafish group that was injected with higher concentration of *lmna* MO (0.5 mm) exhibited a significant drop in their average heart rate (107.8 bpm) mimicking bradycardia. This was similar to the effect of the novel *LMNA* variant, indicating that the variant has a strong effect on LMNA function (Fig. 4B).

### *LMNA* variant expression resulted in morphological defects

**Figure 5:**
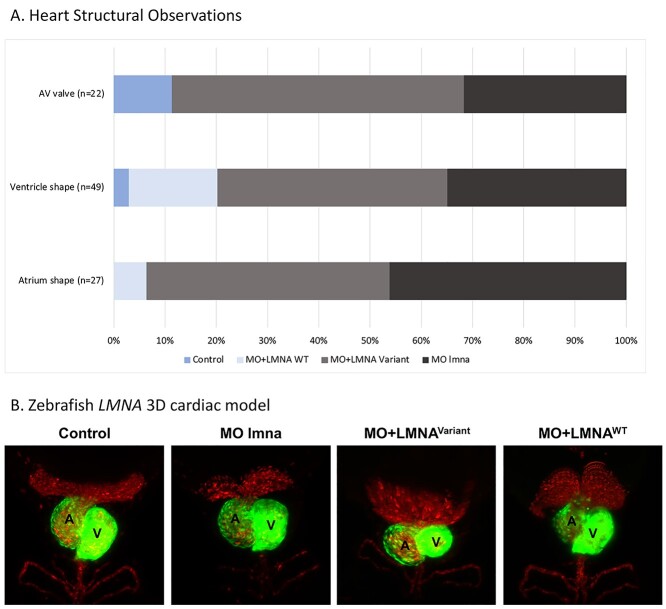
Zebrafish *LMNA* model heart structure abnormalities. Modeling Qatar Genome Programme human *LMNA* cardiac variant produced abnormal heart chambers in *LMNA* zebrafish model. (**A**) Proportion of heart abnormalities, including valve defects and abnormal heart chamber shape in the four experimental groups. (**B**) *LMNA* zebrafish heart 3D structure at 72 hours post fertilization (hpf). Transgenic embryos (Tg:gata1: dsRed, cmlc2:eGFP), expressing red fluorescent red blood cells and green hearts) showing the abnormal cardiac chambers structure (Atrium: A, Ventricle: V). AV valve: Control (n = 3), *lmna* MO (*n* = 5), MO + LMNA^Variant^ (*n* = 14), and MO + LMNA^WT^ (*n* = 0). Ventricular shape: Control (*n* = 2), *lmna* MO (*n* = 14), MO + LMNA^Variant^ (*n* = 28), and MO + LMNA^WT^ (*n* = 5). Atrium shape: Control (*n* = 0), MO (*n* = 10), MO + LMNA^Variant^ (*n* = 16), and MO + LMNA^WT^ (*n* = 1).

Human cardiac laminopathy has been linked to morphological and functional defects (21). Examination of the zebrafish cardiac chambers showed that both the novel *LMNA* variant (MO + LMNA^Variant^) and zebrafish knockdown (*lmna* MO) groups presented with abnormal ventricular morphology compared to the MO + LMNA^WT^ and control groups. Our scoring for heart malformations (Supplementary Material, Fig. S6) showed that the novel *LMNA* variant had a significant association with abnormal atrium shape (*P* < 0.0001), abnormal ventricle shape (*P*-value <  0.0001) and abnormal atrioventricular valve (*P* = 0.0008) (Fig. 5A).

To confirm the structural abnormality in the 3D heart model, we used transgenic embryos (Tg:gata1: dsRed, cmlc2: eGFP), to examine the heart chambers’ structure. We confirmed the pathogenetic impact of the *LMNA* human variant on the zebrafish cardiovascular system (Fig. 5B and Supplementary Material, Videos S13–S16). Our experimental design displayed the effect of the *LMNA* human genetic variation on the cardiac chambers’ structure. We observed abnormal cardiac chambers in the MO + LMNA^Variant^ and *lmna* MO groups.

## Discussion

The integration of genetic data with deep phenotypic analysis and family history is a useful tool to identify individuals at risk for rare genetic diseases. The Middle Eastern, Arab population is characterized by a high consanguinity rate that can reach up to 50% with marriages predominantly between first cousins (22,23). This high rate of consanguineous unions resulted in genetic disorders being one of the leading cause of mortality in the Arabic population (24,25). Many variants are only present or have higher frequency in the Arabic population, therefore it is important to study their potential pathogenic effects. This study expands our knowledge about medically actionable variants in this underrepresented population.

Our filtering criteria for the novel variants with potential pathogenic effect involved computational predictions, allele frequency in the QGP cohort and segregation evidence. However, it is difficult to establish genotype-disease associations for most, because the QGP cohort is a population-based cohort, and the pathogenicity of the variants in the 59 ACMG genes may not present clinically until late adulthood (26). We have recently reported a total of 60 known pathogenic and likely pathogenic variants in 25 ACMG genes in 141 unique individuals using the same data (27). In this study, we found 270 genotype-positive participants for the 74 potentially pathogenic novel variants in the QGP cohort, indicating that an additional 4.5% of the population might have increased risk of developing one of the diseases these genes relate to.

Beyond assessing the general frequency of novel ACMG variants, this study provided insight into the frequency of novel candidate variants among different sub-populations existing in the QGP cohort. We observed heterogeneous distribution of the novel variants among the studied six QGP sub-populations. There were no novel variants found in the smallest SAS sub-population. The highest frequency of novel variants was found in the WEP sub-population, with most variants present in genes associated with cardiovascular diseases and malignant hyperthermia. In general, genes associated with cardiac conditions had the highest variant frequency (28%) in the QGP. Interestingly, this finding is consistent with the high prevalence of cardiovascular diseases in Qatar (28–30).

The *DSP* gene encodes for the structural desmoplakin protein, an important component of the desmosome. Many pathogenic mutations in the *DSP* gene had been reported in patients with arrhythmogenic cardiomyopathy in multiple case series (31–37). Typically, arrhythmogenic cardiomyopathy presents in late adulthood, but also many pediatric cases were reported (38,39).

In this study, we examined the *in vivo* effect of the novel *DSP* variant c.1841A > G (p.Asp614Gly) on the zebrafish cardiac function. Previous phylogenetic studies confirmed that there are two zebrafish orthologs (*dspa* and *dspb*) of human the *DSP* (40). Both orthologs are expressed and can be detected in the cardiac embryonic region of 2 dpf zebrafish. We utilized the transient knockdown of the zebrafish *dspa* and *dspb* genes and co-injection of either of the WT or the variant-carrying synthetic human *DSP* RNA. We observed that both the novel *DSP* variant c.1841A > G (p.Asp614Gly) (MO + DSP^Variant^) and the zebrafish knockdown (*dspa/b* MO) groups elicited negative effects on zebrafish survival and development. This is consistent with the previously reported effect of antisense MO-mediated knockdown of zebrafish *dsp*s (40). Furthermore, embryonic lethality and growth delay were also reported upon homozygous deletion of *Dsp* in mice (41). These effects are mainly due to cell adhesion defects. We only performed the cardiac analyses on apparently normally developed animals to avoid any toxicity or teratogenic effects of the RNA injections.

Both the human *DSP* variant and the zebrafish knockdown resulted in zebrafish cardiac bradycardia. We observed a decrease in the blood flow activity and irregular blood flow rhythm for both the MO + DSP^Variant^ and *dspa/b* MO groups. Similarly, patients with arrhythmogenic cardiomyopathy, usually present with dominant ventricular arrhythmia (42–44). While the diagnosis of arrhythmogenic cardiomyopathy can be challenging due to its heterogeneous presentation and inconsistent ECG findings (37), a recent publication by Liang *et al.* using a large case cohort (*n* = 522) showed that bradyarrhythmia were commonly seen with atrial involvement in patients with definitive diagnosis of arrhythmogenic cardiomyopathy (45). Overall, our results from the *DSP* zebrafish model confirmed that the novel *DSP* variant is pathogenic as it induced an effect on the zebrafish cardiac function that corresponded to the manifestation of human arrhythmogenic cardiomyopathy.

Another important gene for normal heart development and function is *LMNA*. Mutations that disrupt the production of normal lamin proteins that is encoded by the *LMNA* gene cause a group of human disorders collectively called laminopathies (46). One of the major disease types of laminopathies is the involvement of the cardiac striated muscle leading to *LMNA*-related cardiomyopathy (20).

We showed that *lmna* knockdown (*lmna* MO) and co-expression of WT or variant-carrying *LMNA* RNA with the knockdown (MO + LMNA^WT^, MO + LMNA^Variant^) all negatively affected zebrafish survival rate. The *lmna* MO and the MO + LMNA^Variant^ groups had survival rates ~40%, while the MO + LMNA^WT^ showed partial rescue with 65% survival rate. This could partly be explained by the well-known structural role of lamin proteins encoded by the LMNA gene in the nuclear envelope and their role in regulating transcription through modulation of chromatin organization, DNA replication and signal transduction pathways (47,48).

Our zebrafish *LMNA* model showed that the human *LMNA* variant c.326 T > G (p.Val109Gly) resulted in bradycardia and abnormal heart morphology. Interestingly, zebrafish knockdown using two different doses of *lmna* MO (0.35 mm and 0.5 mm) produced tachycardia and bradycardia, respectively. This observation indicates that the effect of a *lmna* knockdown is dose dependent on the zebrafish heart rate with more severe consequences produced by higher *lmna* MO doses, suggesting progression toward heart failure. The effect of the human *LMNA* variant on the zebrafish heart rate resembles the effect of the higher *lmna* MO dose. This severe bradycardia effect in patients with *LMNA*-related cardiomyopathy is indicative of a high degree atrioventricular block and end-stage heart failure (21). Other mutations in the *LMNA* gene and MO mediated zebrafish *lmna* knockdown were reported to produce different heart rate patterns of the zebrafish heart, blood congestion, and heart edema and failure (49–51). Based on these results we conclude that the novel *LMNA* variant resembles the severe pathogenic effect produced by *LMNA*-related cardiomyopathy. A knockdown and splicing approach of *lmna* zebrafish gene by different types of morpholino oligonucleotides to impair the function of lamin A/C proteins was conducted previously (50). The study found that different phenotypes in zebrafish embryos resulted from different morpholinos due to their impact on the cell cycle. Our 3D heart *LMNA* model showed a clear difference in the heart chambers but assessing a larger number of zebrafish samples would be needed to verify a significant result.

Furthermore, our study’s limitations prevented us from assessing the full spectrum of symptoms related to *DSP-* and *LMNA-*mediated cardiomyopathies. *DSP-*related disease leads to late cardiac structural changes, which we could not assess due to the short morpholino activity window, which prevented us from assessing any late structural impact in the zebrafish myocardium. Therefore, alternative approaches should be used for further extensive morphological assessment of late structural changes, as expected in *DSP-*related cardiomyopathy. Additionally, electrocardiographic (ECG) analyses could evaluate the presence of bradyarrhythmia and heart blocks. Since *LMNA*-related cardiomyopathy can result in bradyarrhythmia and heart block (commonly first-degree heart block with the possibility to progress into second/third degree block), this would be required to establish further similarities between the *LMNA-*mediated human disease and the zebrafish model.

In conclusion, our findings demonstrate that there is a comparable number of novel predicted pathogenic variants in the 59 ACMG genes in the Qatari population to the known pathogenic variants in the same genes. Further studies are required to establish firm evidence of pathogenicity for the variants that have not been characterized, and we recommend inclusion of these variants in reporting of incidental findings in individuals of Middle Eastern descent.

## Materials and Methods

### Cohort and sample description

This study was conducted on 6045 QGP and QBB participants. The QBB is a prospective population study aimed to recruit 60 000 participants from the Qatari population aged 18 years or above. Detailed description of the study cohort, phenotypic assessment and sample collection was previously described by Al Thani *et al.* (12). All QBB participants signed a generic consent form prior to their participation that allowed their data to be used anonymously. The QBB study was approved by the Hamad Medical Corporation Ethics Committee and QBB institutional review board (IRB). Our study was registered and approved by QBB under the IRB protocol number, QF-QGP-RES-PUB-002. Whole peripheral blood was collected from each participant enrolled in this study and DNA was extracted as previously described (27,52,53). Samples were sequenced on an Illumina HiSeq X Ten (Illumina, USA) machine to an average sequencing depth of 30× coverage.

### Bioinformatics annotation and filtering criteria of novel variants in 59 ACMG genes

Corresponding to the 59 ACMG genes, 148 285 variants were retrieved from the QGP dataset in the WGS data using the QGP pipeline as previously described (27). Briefly, read QC was performed using FastQC (v0.11.2) (54) reads were mapped to the GRCh37 (hs37d53) version of the human genome by the bwa.kit (v0.7.12) (55). We developed filtering criteria to identify novel variants in 6045 WGS data from the QGP. We retrieved all QGP variants that fell into the coding regions of the ACMG 59 genes and were not reported in the following databases: dbSNP (build 151)***,*** gnomAD Exome and WGS (r2.0.2), 1000 Genome project (56), ExAC (r0.3.1), TOPMed***,*** GME (8), HGMD (v2018.2) and ClinVar (v20190211). We filtered potentially pathogenic novel variants according to CADD score ≥ 20 and GERP ≥ 3. We selected the variants with minor allele frequency (MAF) <0.001 in the QGP cohort. We removed all variants with single allele counts in the QGP data, then selected the variants which were present in at least two unrelated QGP participants. The variants were filtered according to impact (moderate) and effect (intron variants and loss of function variants were removed).

The QGP data set comprised six sub-populations: General Arabs (GAR, *n* = 2311), Peninsular Arabs (PAR, *n* = 1052), Arabs of Western Eurasia and Persia (WEP, *n* = 1372), Arabs with South Asian ancestry (SAS, *n* = 38), Arabs with African ancestry (AFR, *n* = 92) and Admixed (ADM, *n* = 1180) as described previously (57). We compared the allele frequency of the filtered novel variants across the sub-populations in the QGP data set. Additionally, we evaluated the allele frequencies of filtered novel variants across different disease categories of 59 ACMG genes.

### Selection of cardiovascular variants for functional analysis in zebrafish

From the phenotypic data available from the QBB repository, we extracted those related to cardiovascular diseases (ECG, family history and self-reported information about heart diseases). We established the following segregation criteria to select the novel genetic variants, which are predicted to be pathogenic for functional validation in zebrafish: The variants should show significant association with cardiovascular disease, defined as abnormal ECG report or borderline ECG report and self-reported data of cardiovascular symptoms. At least two genotype-positive participants should have cardiovascular disease. Additionally, genotype-positive participants for selected variants should have positive parent history of cardiovascular diseases. Participants who were found to carry known pathogenic variants in genes related to cardiovascular diseases were excluded. Furthermore, we used bioinformatic tools, MutationTaster (58) and I-Mutant2.0 (59) to predict the effect of the selected variants for their disease-causing potential and the effect of the genetic variation on protein stability.

### Zebrafish care and maintenance

We used the Pentair Aquatic Habitats system to maintain the zebrafish. The system provides 14 h and 10 h cycles of on and off light, respectively. The temperature in the system was held to be 28°C. *Artemia nauplii* was used as a source of food for zebrafish. We used a high dose of Tricaine MS-222 (200 mg/L) followed by ice for euthanizing the zebrafish.

The local Animal Care and Use Committee approved protocols used in this experiment. Additionally, all zebrafish protocols have adhered to the policy presented by the Qatar Ministry of Public Health, which conforms to the National Institutes of Health guidelines for the Care and Use of Laboratory Animals. All zebrafish experiments conducted in this study were following IACUC Office in Qatar foundation, protocol EVMC-2020-006.

### Phylogenetic analysis

Desmoplakin protein sequences encoded by the *DSP* gene were extracted from Ensemble (longest isoforms were selected) for human DSP protein (NP_001008844.1), Zebrafish Dspa (XP_005171161.1), Zebrafish Dspb (XP_021324397.1) and other species; Rat DSP (XP_001058477.1), Mouse DSP (NP_076331.2) and Chicken DSP (XP_418957.3), to investigate the conservation of the human *DSP* variant c.1841A > G (p.Asp614Gly) across the different species. Similarly, Lamin A/C sequences were extracted for the human LMNA protein (NP_733821.1), Zebrafish Lmna (NP_694503.1) and other species; Rat LMNA (NP_001002016.2), Mouse LMNA (NP_001002011.2) and Chicken LMNA (NP_990618.1), for the human *LMNA* variant c.326 T > G (p.Val109Gly). The multiple alignments were performed using the CLC Sequence Viewer algorithm (v.7).

### Design of Morpholino antisense oligos and zebrafish microinjection

We designed MO for *in vivo* knockdown of the zebrafish *dspa*, *dspb* and *lmna* (Gene Tools). MO sequences targeting the zebrafish *dspa*-201 ENSDART00000148460.4 transcript translation start site was: 5′-GGTCTGAGAACCGGACAAACTCATC-3′, and for the *dspb*-201 ENSDART00000111078.4 transcript, targeting its exon4 -intron4 boundary was 5’-TCTGACTGTGTTTCAGACTGACCTG-3′. For the zebrafish *lmna*-204 ENSDART00000184045.1 transcript, we used translation blocking MO: 5′- CATGGTTGTCTGGAACTACTGATAA-3′. The control mispair MO sequence was: 5′-CCTCTTACCTCAGTTACAATTTATA-3′. Microinjection of 1–2 nL was performed into single-cell zebrafish embryo using PLI-100 Picolitre injector, Harvard Apparatus as described previously (60).

### Human *DSP* and *LMNA* synthetic RNA rescue experiments

Human *DSP* cDNA (NM_001008844.3) and human *LMNA* cDNA (NM_170707.4) were cloned into the pBluescript-II-KS+ vector (Genescript). DSP_OHu101428C_pBluescript II KS(+) and LMNA_OHu21250C_pBluescript II KS(+) were linearized by restriction digest at the 3′ (restriction enzyme KpnI, New England Biolabs, Cat#: R0142S) and used as a template to synthesize the human *DSP* mRNA and *LMNA* mRNA using the mMESSAGE mMACHINE T7 Ultra transcription kit (Invitrogen, Cat#: AM1345). Equal amounts of WT human *DSP* and *LMNA* synthetic mRNA were co-injected with MO with rhodamine stain (Cat#: PLAZM0071) against the two variants as previously described (61). The examined zebrafish groups were human WT and variant *DSP* mRNA co-injected with *dspa* + *dspb* MO (MO + DSP^WT^ and MO + DSP^Variant^), and human WT and variant *LMNA* mRNA co-injected with *lmna* MO (MO + LMNA^WT^ and MO + LMNA^Variant^) compared to control uninjected and control MO injected groups (*dspa/b* MO, *lmna* MO). Dose titrations of the mRNA and MO concentrations were performed. The *dspa* MO and *dspb* MO (0.5 mm) were co-injected with human *DSP* mRNA (150 ng/uL) and *lmna* MO (0.35 mm) was co-injected with human *LMNA* mRNA (200 ng/uL). At least 50 embryos were injected in each group for three sets of experiments.

### Quantitative gene expression analysis of Desmoplakin and Lamin a/C

To assay the knockdown of endogenous zebrafish *dspa/b* and *lmna* expressions using *dspa* + *dspb* MO and *lmna* MO, respectively, total RNA was extracted from embryos of the MOs and controls (uninjected). Total RNA was isolated using TRIZOL reagent (life technologies, Carlsbad, California, United States) and purified using RNeasy mini kit (Qiagen) according to manufacturer’s recommendation. First Strand cDNA Synthesis was performed using reverse transcriptase Kit, superscript III (Invitrogen) from 500 ng RNA. The resulting cDNA was used for quantitative PCR (RT-qPCR), a triplicate of the synthesized cDNA was amplified using SYBR select master mix (Life Technologies). Gene-specific primers were used for amplification of the PCR and eukaryotic elongation factor (EF1A) was used as a housekeeping gene for normalization. The primers used for each gene were *lmna*: (forward: 5’-TTTGTTCCAGACCACCCTCATC-3′, reverse: 5’-AAGCACAGATGGTCAGGGTTTG-3′), *dspa*: (forward: F 5’-ACCAAACATCTGCACGTTCC-3′, reverse: R 5’-CAGGTATCGGCTCTGCTCAG-3′) and *dspb*: (forward: 5’-ATGCTGAACGAACTCAACGC-3′, reverse: 5’-TATCTCTGCACTTCCTCCGG-3′). For the EF1a we used (forward: 5’-ACCACCGGCCATCTGATCTACAAA-3′, and the reverse primer: 5’-ACGGATGTCCTTGACAGACACGTT-3′).

### Gross morphological examination of zebrafish groups

The survival rates of the different zebrafish groups were assessed at 24 hours post fertilization (hpf). The classification of the zebrafish development was carried out at 72 hpf, prior to imaging. We proceeded to subsequent analyses using only apparently normally developed animals (G3) to avoid non-specific effects due to the RNA-injections. The cardiac phenotypes at 72 hpf were examined by video recordings using a Stereomicroscope Zeiss LUMAR.V12 with Plan Apo S 1.5× objective. We used Micro-Manager and ImageJ (BSD-2) to evaluate the zebrafish heart function. Using 1920 × 1080 resolution of the monochrome camera (model DMK 33UX252), zebrafish embryos were captured by time-lapse videos in 60 frames per second (fps) at 150× magnification. Each zebrafish group was placed in a separate well and kept in 28°C incubator until examination. Afterward, zebrafish larvae were stabilized in methylcellulose 2.5% (Sigma, Cat# M0387), and images were taken using a stereomicroscope with 28°C heated stage glass.

### Analysis of heart morphology, heart rate and flow rate

To analyze the functional impact of the human genetic variants, zebrafish at 72 hpf were examined. Heart development is complete at 48 hpf (62). Recorded videos (Supplementary Material, Videos S1–S8) were imported into the DanioScope software (Noldus, version 1.0.109) as uncompressed AVI files, where subsets of the beating heart chambers were outlined, and the heart rate was calculated. To calculate the flow activity percentage in the zebrafish Dorsal Aorta (DA), video recordings (Supplementary Material, Videos S9–S12) were captured at 60 fps of the caudal area. Using the Danio scope software, an area was selected to measure the flow rate activity as previously described (63).

### Heart 3D structure of zebrafish *LMNA* variant model

Transgenic embryos (Tg:gata1: dsRed, cmlc2: eGFP), expressing red fluorescent red blood cells and green hearts, were examined for the cardiac chambers’ structure (atrium and ventricle).

### Statistical analysis

All results were presented as means of at least three experiments. The genotype–phenotype association significance was calculated using Fisher’s exact test. Chi-square test was used to compare the survival rate and the frequency of normally developed groups. Multiple comparisons to the control, to compare the heart rate and the flow activity for zebrafish *DSP* and *LMNA* models were analyzed by *t*-tests. All statistical tests were calculated using GraphPad Prism (version 8.4.3). The level of significance was expressed using *P*-values: ^*^P < 0.05, ^*^^*^P < 0.01, ^*^^*^^*^P < 0.001, ^*^^*^^*^^*^P < 0.0001.

## Data Availability

The data that support the findings of this study are available from the QGP and QBB but restrictions apply to the availability of these data, which were used under license QF-QGP-RES-PUB-002 for the current study, and so are not publicly available. Data are however available from the authors upon reasonable request and with permission of QBB institutional review board.

## Authors’ Contributions

A.E. and B.M. were involved in conceptualization. A.E. and S.D. performed the experiments. A.E. and H.M. analyzed the phenotypic information for the participants. A.E., D.A., I.M. and W.H. contributed to the zebrafish experiments and analysis. A.E., S.D. and B.M. wrote the main manuscript text. All authors read and approved the final manuscript.

*Conflict of Interest statement*. None declared.

## Competing Interests

The authors declare that they have no competing interests.

## Ethics Approval and Consent to Participate

All QBB participants signed a generic consent form prior their participation that allowed their data to be used anonymously. The QBB study was approved by Hamad Medical Corporation Ethics Committee and QBB institutional review board (IRB). To access the WGS data and phenotypic data, a request was applied to QBB which was approved by the QBB IRB (IRB protocol number, QF-QGP-RES-PUB-002).

All protocols used in this study were approved by the local Animal Care and Use Committee and conform to the Zebrafish Policy published by the Qatar Ministry of Public Health that follows the Guide for the Care and Use of Laboratory Animals published by the National Institutes of Health. Experiments performed on zebrafish followed Qatar Foundation, IACUC Office, regulations under Protocol Number: EVMC-2020-006.

## The Qatar Genome Program Research Consortium

Qatar Genome Project Management: Said I. Ismail^1^, Wadha Al-Muftah^1^, Radja Badji^1^, Hamdi Mbarek^1^, Dima Darwish^1^, Tasnim Fadl^1^, Heba Yasin^1^, Maryem Ennaifar^1^, Rania Abdel-latif^1^, Fatima Alkuwari^1^, Muhammad Alvi^1^, Yasser Al Sarraj^1^, Chadi Saad^1^, Asmaa Althani^1,2^.

Biobank and Sample Preparation: Eleni Fthenou^2^, Fatima Qafoud^2^, Eiman Alkhayat^2^, Nahla Afifi^2^.

Sequencing and Genotyping group: Sara Tomei^3^, Wei Liu^3^, Stephan Lorenz^3^.

Applied Bioinformatics Core: Najeeb Syed^4^, Hakeem Almabrazi^4^, Fazulur Rehaman Vempalli^4^, Ramzi Temanni^4^.

Data Management and Computing Infrastructure group: Tariq Abu Saqri^5^, Mohammed Husen Khatib^5^, Mehshad Hamza^5^, Tariq Abu Zaid^5^, Ahmed El Khouly^5^, Tushar Pathare^5^, Shafeeq Poolat^5^, Rashid Al-Ali^5^.

Consortium Lead Principal Investigators: Omar M. E. Albagha^6^, Souhaila Al-Khodor^7^, Mashael Alshafai^8^, Ramin Badii^9^, Lotfi Chouchane^10^, Xavier Estivill^11^, Khalid A. Fakhro^12^, Hamdi Mbarek^1^, Younes Mokrab^13^, Jithesh V. Puthen^6^, Karsten Suhre^14^, Zohreh Tatari^15^

1. Qatar Genome Program, Qatar Foundation, Qatar Science and Technology Park, Innovation Center, Doha, Qatar.
2. Qatar Biobank for Medical Research, Qatar Foundation, Building 317, Hamad Medical City, Doha, Qatar.
3. Sidra Medicine, Integrated Genomics Services, Out-Patient Clinic, Doha, Qatar.
4. Sidra Medicine, Applied Bioinformatics Core—Integrated Genomics Services—Research Branch, Doha, Qatar.
5. Sidra Medicine, Biomedical Informatics—Research Branch, Doha, Qatar.
6. College of Health and Life Sciences, Hamad Bin Khalifa University, Education City, Doha, Qatar.
7. Sidra Medicine, Maternal and Child Health Program, Doha Qatar.
8. College of Health Sciences, Qatar University, Doha, Qatar.
9. Molecular Genetics Laboratory, Hamad Medical Corporation, Doha, Qatar.
10. Departments of Genetic Medicine, Microbiology and Immunology, Weill Cornell Medicine-Qatar, Doha, Qatar.
11. Quantitative Genomic Medicine Laboratories (qGenomics), Barcelona, Spain.
12. Sidra Medicine, Human Genetics Department, Doha, Qatar.
13. Sidra Medicine, Computational Genomics and Data Science Laboratory, Doha, Qatar.
14. Bioinformatics Core, Weill Cornell Medicine-Qatar, Education City, Doha, Qatar.
15. Sidra Medicine, Clinical Research Center, Doha, Qatar.

## Supplementary Material

Additional_File_1_ddac073

Supplemental_Tables_ddac073

Additional_File_3_ddac073
