# Supplementary material for: Analysis of incidental findings in Qatar genome participants reveals novel functional variants in LMNA and DSP
Source: Hum Mol Genet. 2022 Mar 26;31(16):2796–809. doi: 10.1093/hmg/ddac073 (PMC9402234; doi:10.1093/hmg/ddac073)
Supplement: Additional_File_1_ddac073 [file additional_file_1_ddac073.docx]

**Supplemental Figures**

**Supp. Figure S1: Filtering criteria of novel variants in 59 ACMG genes from QGP 6,045 WGS data.** 148,285 variants in the ACMG 59 genes retrieved and filtered from 6,045 WGS data from QGP. Abbreviations: QGP; Qatar Genome Program, V; variants, MAF; minor allele frequency, AC; allele count.

**Supp. Figure S2: Human DSP and LMNA missense variants are characterized in zebrafish model using a morpholino targeting its zebrafish ortholog.** (A) Alignment between human DSP protein (NP_001008844.1) sequence and its zebrafish orthologs dspa (XP_005171161.1) and dspb (XP_021324397.1), showing the amino location of the human variant (red asterisk). Schematic representation of the Human DSP modeled by Morpholino (MO) targeting zebrafish dspa ATG translation site and dspb exon / intron 4 splice junction site. Co-injections of MO mix and synthetic human DSP RNA of wildtype (MO+WT) and variant (MO+Variant) (c.1841A>G). Gel electrophoresis showing bands size of *DSP* gene (Var=*DSP* Variant). (B) Alignment between human LMNA protein (NP_733821.1) sequence and its zebrafish ortholog Lmna (NP_694503.1), showing the amino location of the human variant (green asterisk). Schematic representation of the Human *LMNA* modeled by morpholino (MO) targeting zebrafish *lmna* ATG translation site. Co-injections of MO and synthetic human *LMNA* RNA of wildtype (MO+WT) and variant (MO+Variant) (c.326T>G). Human synthetic RNA production using linearized pBluescript II KS(+) vector to in vitro transcribe the RNA using mMessage T7 transcription kit. Gel electrophoresis showing bands size of *LMNA* gene (Var=*LMNA* Variant).

**Supp. Figure S3: Quantitative gene expression analysis of zebrafish *dspa/b* and *lmna* knockdown.** (A). *dspa/b* morpholinos (*dspa* MO, and *dspb* MO) resulted in a significant decrease in endogenous *dspa/b* zebrafish gene expression compared to uninjected control group. (B). *lmna* morpholino (*lmna* MO) resulted in a significant decrease in endogenous zebrafish *lmna* gene expression compared to uninjected control group.

**Supp. Figure S4: Human Qatar Genome Project *DSP* and *LMNA* variants validations in the zebrafish model.** Modeling of Qatar Genome Project human *DSP* and *LMNA* variants in the zebrafish model using morpholino and synthetic human RNA co-injections with rhodamine stain (cat#PLAZM0071): (A) Injected zebrafish embryo at 1-cell stage for the different examined groups with rhodamine red fluorescent stain for screening. Images of cell divisions at 1, 2, and 16-cells stages using fluorescent Zeiss Lumar 12 microscope at 63X magnification. (B) Survival rate for *DSP* zebrafish model. The different groups examined were a set of 3 experiments. MO and human variant had a negative effect on survival rate, (59%, 58%), respectively, compared to control and human wildtype (WT) groups (90%, 81%), respectively. (C) Survival rate for *LMNA* zebrafish model and development effect distribution. The different groups examined were a set of 3 experiments. MO, human variant (MO+Variant) and WT (MO+WT) had a negative effect on survival rate (37%,40%,65%), respectively, compared to control group (90%). D. The effect on development was classified, the distribution between larvae differs in MO injected and MO+Variant in comparison to MO+WT and control groups. G1: severely affected development, G2: mildly affected development, G3: Normal development.

**Supp. Figure S5: Zebrafish heart rate analysis at 72 hours post fertilization (hpf) of control Morpholino in *DSP* and *LMNA* Models.** Zebrafish cardiac heart rate was assessed for control morpholino (MO) (0.125mM), for both MO *dspa/dspb* and MO *lmna* compared to uninjected control. Cardiac video recordings of 6 seconds were analyzed using Danioscope software, the software detects changes in pixel density associated with cardiac muscle contraction and chamber filling and registers the contractions as beats per minute (BPM). Control MO showed no altered effect on the zebrafish heart rate compared to control. Control (n=32), Control MO (n=28). ns: not significant.

**Supp. Figure S6: Representative images of the zebrafish heart at 72 hpf.** (A). Diagram of zebrafish anatomy. A representative image of a transparent, 72 hpf larvae captured with Stereomicroscope Zeiss LUMAR.V12 with Objective Plan Apo S 1.5× at a magnification of 32× using a monochrome camera (The Imaging Source) at 1920×1080 resolution. Organs and anatomical features are denoted in the figure. (B). Schematic illustration of a normal zebrafish heart, showing the direction of blood flow in one cardiac cycle. (C). Representative image of normal heart shape in zebrafish larvae at 72 hpf. Atrium (A), Ventricle (V). (D). Representative image of abnormal Atrium shape in zebrafish larvae at 72 hpf, showing the looping and abnormal shape. (E). Representative image of abnormal ventricle shape in zebrafish larvae at 72 hpf. (F). Representative image of abnormally widened atrioventricular valve in zebrafish larvae at 72 hpf.
