# Supplementary material for: Analysis of incidental findings in Qatar genome participants reveals novel functional variants in LMNA and DSP
Source: Hum Mol Genet. 2022 Mar 26;31(16):2796–809. doi: 10.1093/hmg/ddac073 (PMC9402234; doi:10.1093/hmg/ddac073)
Supplement: Additional_File_3_ddac073 [file additional_file_3_ddac073.docx]

**Supplemental Videos**

**Supp. Videos S1-8. QGP *DSP* and *LMNA* human variant in zebrafish model.**

**Supp. Videos S9-12. QGP *DSP*, Dorsal Aorta blood flow pattern in zebrafish model.**

**Supp. Videos S13-16. QGP *LMNA* human variant displayed variable cardiac phenotypes in the zebrafish 3D cardiac models.**

Link of Supplemental Videos: <https://utshbku.egnyte.com/fl/HB8PaKuvcX>
